# White matter changes in fetal brains with ventriculomegaly

**DOI:** 10.3389/fnana.2023.1160742

**Published:** 2023-06-14

**Authors:** Bianca Horgos, Miruna Mecea, Armand Boer, Andrei Buruiana, Razvan Ciortea, Carmen-Mihaela Mihu, Ioan Stefan Florian, Alexandru Ioan Florian, Florin Stamatian, Bianca Szabo, Camelia Albu, Sergiu Susman, Raluca Pascalau

**Affiliations:** ^1^Faculty of Medicine, “Iuliu Haţieganu” University of Medicine and Pharmacy, Cluj-Napoca, Romania; ^2^Department of Oncology, “Ion Chiricuţă” Institute of Oncology, Cluj-Napoca, Romania; ^3^Department of Obstetrics and Gynecology, “Iuliu Haţieganu” University of Medicine and Pharmacy, Cluj-Napoca, Romania; ^4^Department of Obstetrics and Gynecology, Emergency County Hospital, Cluj-Napoca, Romania; ^5^Department of Morphological Sciences—Histology, “Iuliu Haţieganu” University of Medicine and Pharmacy, Cluj-Napoca, Romania; ^6^Department of Neuroscience—Neurosurgery, “Iuliu Haţieganu” University of Medicine and Pharmacy, Cluj-Napoca, Romania; ^7^Department of Neurosurgery, Emergency County Hospital, Cluj-Napoca, Romania; ^8^Department of Obstetrics and Gynecology, IMOGEN Centre of Advanced Research Studies, Cluj-Napoca, Romania; ^9^Department of Morphological Sciences—Anatomy and Embryology, “Iuliu Haţieganu” University of Medicine and Pharmacy, Cluj-Napoca, Romania; ^10^Department of Morphological Sciences—Pathology, “Iuliu Haţieganu” University of Medicine and Pharmacy, Cluj-Napoca, Romania; ^11^Department of Pathology, IMOGEN Centre of Advanced Research Studies, Emergency County Hospital, Cluj-Napoca, Romania; ^12^Department of Ophthalmology, Emergency County Hospital, Cluj-Napoca, Romania; ^13^Research and Development Institute, Transilvania University of Brasov, Brasov, Romania

**Keywords:** fetal brain, white matter tracts, fiber dissection, human brain development, ventriculomegaly

## Abstract

**Introduction:**

Ventriculomegaly (VM) is a fetal brain malformation which may present independently (isolated form) or in association with different cerebral malformations, genetic syndromes or other pathologies (non-isolated form).

**Methods:**

This paper aims to study the effect of ventriculomegaly on the internal tridimensional architecture of fetal brains by way of Klingler's dissection. Ventriculomegaly was diagnosed using fetal ultrasonography during pregnancy and subsequently confirmed by necropsy. Taking into consideration the diameter of the lateral ventricle (measured at the level of the atrium), the brains were divided into two groups: moderate ventriculomegaly (with atrial diameter between 13 and 15 mm) and severe ventriculomegaly (with atrial diameter above 15 mm).

**Results and discussion:**

The results of each dissection were described and illustrated, then compared with age-matched reference brains. In the pathological brains, fascicles in direct contact with the enlarged ventricles were found to be thinner and displaced inferiorly, the opening of the uncinate fasciculus was wider, the fornix was no longer in contact with the corpus callosum and the convexity of the corpus callosum was inverted. We have studied the prevalence of neurodevelopmental delay in children born with ventriculomegaly in the literature and discovered that a normal developmental outcome was found in over 90% of the mild VM cases, approximately 75% of the moderate and 60% in severe VM, with the correlated neurological impairments ranging from attention deficits to psychiatric disorders.

## Introduction

Ventriculomegaly (VM) is the term generally used to refer to a fetal enlargement of the ventricular system (Pisapia et al., 2017). This can be the result of hydrocephalus, brain dysgenesis, or atrophy. Hydrocephalus describes the active distension of the ventricular system due to anomalies in cerebrospinal fluid (CSF) production and drainage, which lead to inappropriate flow (Rekate, 2008). The CSF is mainly produced by the choroid plexus and flows through the ventricular system toward the subarachnoid space, from where it is drained into the superior sagittal sinus via the arachnoid granulations (Bothwell et al., 2019). VM is a relatively common finding on prenatal screening, observed in ~1% of pregnancies (Salomon et al., 2007; Giorgione et al., 2022), thus being the most common intracranial abnormality detected antenatally (Griffiths et al., 2022). Based on the presence or absence of other abnormalities these cases can be roughly divided into isolated VM and non-isolated VM. On the matter of which of the two forms is more frequent, the current literature is inconclusive due to differences in population, classification criteria, and imaging techniques (Weichert et al., 2010; Lipa et al., 2019). Hence, isolated VM is estimated between 33 and 61% among all VM cases (Kelly et al., 2001). In terms of etiology, non-isolated VM is often associated with additional obstructive anomalies such as neural tube defects, Dandy-Walker or Arnold-Chiari type II malformations, as well as non-obstructive developmental disorders such as callosal agenesis or neural migration/proliferation defects (Rickard et al., 2006; Gaglioti et al., 2009). Other causes which accompany VM are aneuploidy/genetic syndromes, infections, infarction, intracerebral hemorrhages, and tumors (Bothwell et al., 2019). In contrast isolated VM is a diagnosis of exclusion, and the etiology is poorly understood (Guibaud and Lacalm, 2015). Still, some authors include in this category single gene disorders (*L1CAM, AP1S2, CCDC88C*, and *MPDZ*) associated with hydrocephalus (Etchegaray et al., 2020), and numerous CNVs were recently identified in isolated VM (Hu et al., 2017).

The outcome of isolated VM is better than the non-isolated forms and depends on ventricular dimensions and disease progression (Wax et al., 2003). Giorgione et al. (2022) indicated a survival rate of 97–98% in cases of isolated mild VM, with an over 90% rate of normal neurodevelopment (Gaglioti et al., 2005; Sethna et al., 2011). In contrast, cases of severe VM, have a survival rate estimated at about 88%, with a 60% chance of neurologic, motor, and cognitive impairment (Carta et al., 2018). From this perspective, aspects concerning the brain architecture in VM are crucial in order to provide pertinent explanations for the neurodevelopmental outcome.

In both isolated and non-isolated VM, the diagnosis is established based on an atrial diameter >10 mm following prenatal US and/or MRI (Bothwell et al., 2019). Fetal MRI is mostly employed after the ultrasonographic diagnosis of ventriculomegaly, for a more detailed view of the brain parenchyma and for the assessment of associated brain anomalies. A study from 2010 discovered, by means of fetal MRI, 25 out of 147 brains with additional associated malformations, especially corpus callosum agenesis, that were initially labeled as isolated VM cases by ultrasonographic assessment alone (Griffiths et al., 2010). A recent MRI based study of VM showed that this pathology is invariably associated with abnormal sulcal developmental patterns (Tarui et al., 2023) indicating probable association of underlying white matter development. Unfortunately, maternal and fetal movement during the procedure, cost and availability are major impediments to acquiring precise information through these MRI techniques (Cardoen et al., 2011), so detailed descriptions of white matter development in the context of VM were not reported using imaging results. For this reason, anatomical studies using Klingler's dissection bring valuable knowledge to the current understanding of fetal ventriculomegaly.

In a previous study (Horgos et al., 2020), using the Klingler technique on healthy fetal brains, we have established that the major white matter tracts emerge in four waves of development. In the first wave (13 GW), the corpus callosum, fornix, anterior commissure, and uncinate fasciculus can be identified. The second wave (14 GW) includes the superior and inferior longitudinal fasciculi and the cingulum. In the third wave (17 GW), the internal capsule emerges. Finally, in the fourth wave of development (20 GW), all the major tracts, including the inferior-occipital fasciculus, can be observed.

In the present study, the main goal was to assess the tridimensional architecture of the fetal white matter under pathological conditions. The internal cerebral structure was examined by the Klingler dissection technique, and the distortions of the white matter tracts were observed and described for each case individually.

## Materials and methods

### Fetal brains

The fetuses included in our study were collected from the Gynecology I and Gynecology II clinics, part of the Clinical County Emergency Hospital Cluj-Napoca, Romania. All fetuses identified with ventriculomegaly among those collected between 1 January 2018 and 31 December 2020 were considered in the current study. Specimens with associated brain pathology (e.g., corpus callosum agenesis and Down's syndrome) were excluded from the study.

The study was conducted on six pathologic brains collected from fetal necropsies, performed by pathology specialists in accordance with standard protocols for intrauterine death diagnostics. The gestational age was calculated based on the reported last day of menstruation and fetal ultrasonographic measurements performed by obstetricians during the pregnancy. All six brains aged in the middle range of the gestational period between 17 and 22 gestational weeks (Table 1). The clinical data corresponding to each patient is summarized in Table 2. The diagnosis of ventriculomegaly was based on fetal ultrasonographic evaluations and confirmed by necropsy. The grade of VM was quantified by measurements of the diameter of the lateral ventricle at the level of atria [Society for Maternal-Fetal Medicine (SMFM) et al., 2018], categorizing our cohort into two groups—moderate and severe VM cases. VM was not detected in all fetuses antenatally. In some cases, abortion was performed for other reasons, and ventricular dilatation was discovered incidentally during necropsy (Patients 2 and 5).

**Table 1:** General measurements of the fetuses at autopsy: length, weight, cranial circumference, and diameters of the anterior and posterior fontanelles.

**Patient's number/ID**	**Gestational age (GW)**	**Fetus**		**Cranium**		
		**Weight**	**Length**	**Cranial circumference**	**Anterior fontanelle**	**Posterior fontanelle**
Patient 1	17	144 g	14 cm	15 cm	2.5/2 cm	1/1 cm
Patient 2	18	231 g	16.5 cm	16 cm	4.5/4 cm	2/1 cm
Patient 3	19	548.35 g	20.5 cm	28.2 cm	1.5/2 cm	0.2/0.2 cm
Patient 4	19	241 g	16 cm	15.5 cm	1/0.5 cm	1/1 cm
Patient 5	21	350 g	17.5 cm	19 cm	3/2 cm	0.5/0.5 cm
Patient 6	22	598 g	20.5 cm	22 cm	2.5/2 cm	1.5/1 cm

**Table 2:** Clinical data of the patients. Associated pathologies of the fetus and the mother, smoking status, and reason for abortion.

**Patient ID**	**Associated fetal pathology**	**Associated pathology of the mother**	**Smoker mother**	**Reason for abortion**
Patient 1	• Craniofacial dysmorphism • Position anomalies at the upper and lower limbs	Hypothyroidism	yes	Increased number of malformations
Patient 2	• Craniofacial dysmorphism • Meckel diverticulum	Lumbar discopathy and radiculopathy	No	Teratogenic risk of the medication
Patient 3	• Macrocephalus • Spina bifida • Lumbar kyphosis • Varus equine of the lower limbs bilaterally • Intrauterine growth restriction	No	No	Increased number of malformations
Patient 4	• Spina bifida with myeloschisis • Chiari II malformation • Omphalomesenteric duct remnants	No	No	Increased number of malformations
Patient 5	Unilateral polymicrogyria	No	Yes	Spontaneous abortion
Patient 6	Meckel diverticulum	No	No	Ventriculomegaly

The brains were collected in the first 1–3 h after the extraction of the fetuses, and preserved in a 4% formaldehyde solution by means of intravascular perfusion and postfixation.

The study protocol was approved by the Ethics Committee of our University.

### White matter dissection

Tridimensional white matter dissections were performed following a stepwise protocol previously adapted by our group for fetal brains (Horgos et al., 2020). In brief, the dissection began with a section along the midline, separating the brain hemispheres. The medial cortex was removed, and the cingulum was identified and removed to expose the underlying corpus callosum. The hemisphere was flipped to its lateral surface, and the lateral cortex was removed. The long association fascicles were dissected and extracted layer by layer in latero-medial succesion. The white matter/gray matter layers underlying the insular cortex were gradually dissected, and the fronto-occipital fasciculus was exposed and removed, revealing the lateral surface of the internal capsule. The hemisphere was flipped again for the dissection of the pillars of the fornix and the anterior commissure. Next, the frontal horn of the lateral ventricle was opened, and the temporal horn of the ventricle was also opened. The fornix, hippocampus, and caudate nucleus were removed so that the medial surface of the internal capsule was reached.

**Figure 1:**
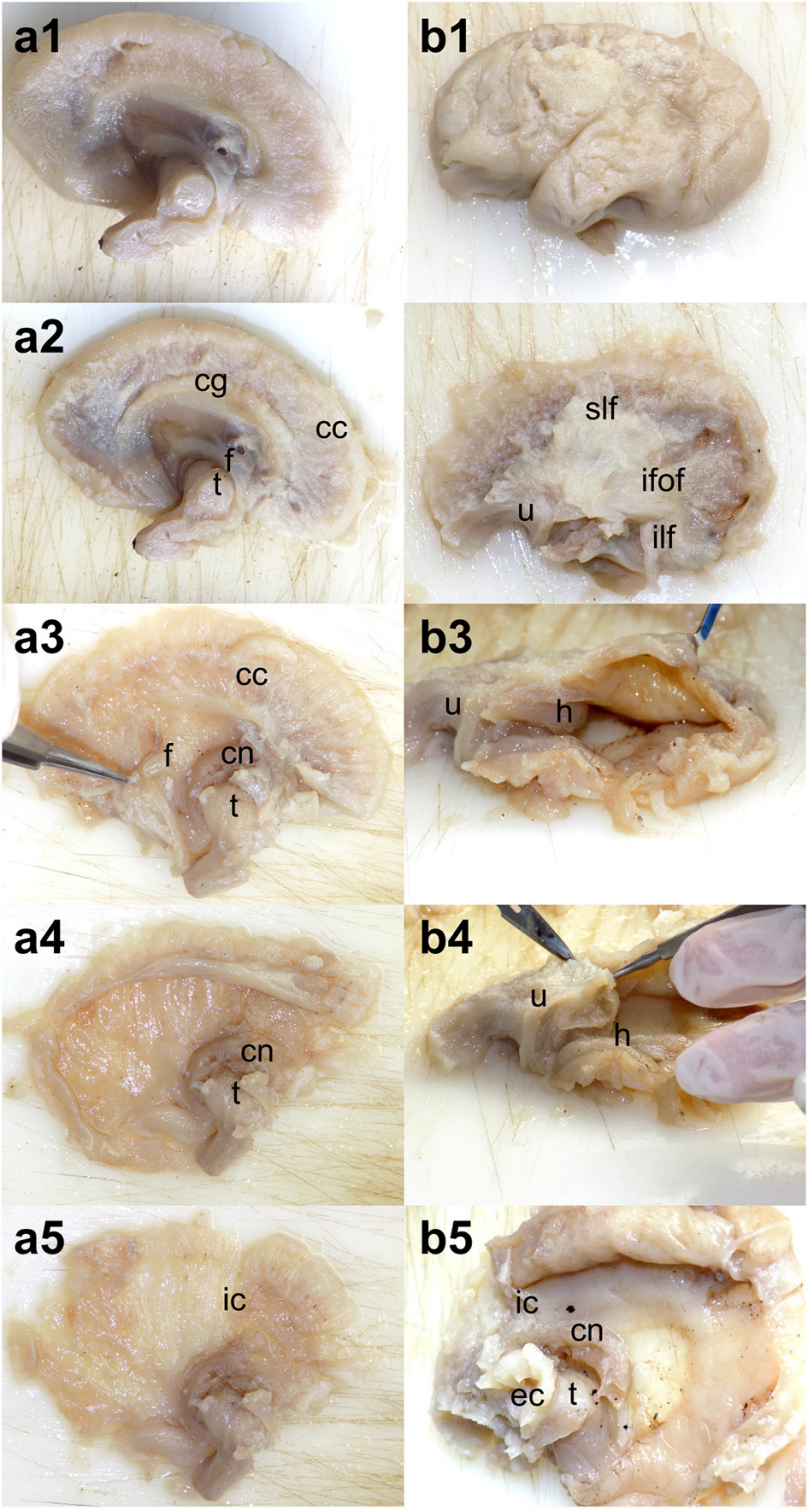
Dissection steps adapted for severe hydrocephalus. **(a1–a5)** Medial to lateral dissection of the white matter. After the removal of the cortex and cingulum, the lateral ventricle was opened from the edges. **(b1–b5)** Lateral to medial dissection of the white matter. The dissection followed the standard protocol through the exposure of the long association tracts but continued with opening the temporal horn of the lateral ventricle followed by the occipital and frontal ones. cg, cingulum; cc, corpus callosum; f, fornix; h, hippocampus; cn, caudate nucleus; t, thalamus; ic, internal capsule; slf, superior longitudinal fasciculus; ilf, inferior longitudinal fasciculus; u, uncinate fasciculus; ifof, inferior fronto-occipital fasciculus; ec, external capsule.

Attempts were made with respect to each step of the protocol—which included dissection of the following commissural (the corpus callosum, anterior commissure, and fornix), association (superior longitudinal, inferior longitudinal, uncinate, inferior fronto-occipital fasciculi, and cingulum), and projection tracts (internal capsule) except for instances in which some of the steps could not be performed due to the tracts being compacted into one another. In some cases, the ventricular cavities were enlarged to such extent that the brain parenchyma had been reduced to a thin lamina through which the ventricles could be seen by transparency. In these cases, following the removal of the cerebral context in the usual fashion the ventricles were opened on the edges, and the white matter was inspected from the inner sides of the ventricles (Figure 1).

## Results

As an observed general statement, the volume of the brain was smaller than that of their healthy counterparts (Horgos et al., 2020). Their external aspect was more globular, and the sulci were shallower, especially the calcarine fissure and the posterior part of the cingulate sulcus. The Sylvian fissure's appearance resembled that of healthy brains.

Regarding the white matter tract, the following changes in their anatomy were discovered to have occurred: (1) the dorsal surface of the corpus callosum was convex instead of concave, (2) the body of the fornix was no longer in contact with the corpus callosum, (3) the uncinate fasciculus was pushed inferiorly with its opening widened, (4) the inferior longitudinal fasciculus was displaced inferiorly and appeared thinner, and (5) the association tracts of the lateral portion of the hemisphere and the internal capsule were compacted into one another, rendering their separation very difficult. Therefore, some tracts could not be identified by dissection at the age at which they had been identified on healthy brains. This does not necessarily imply that the development of the tracts had been delayed. The cingulum and the anterior commissure were found to be least affected among the tracts.

**Figure 2:**
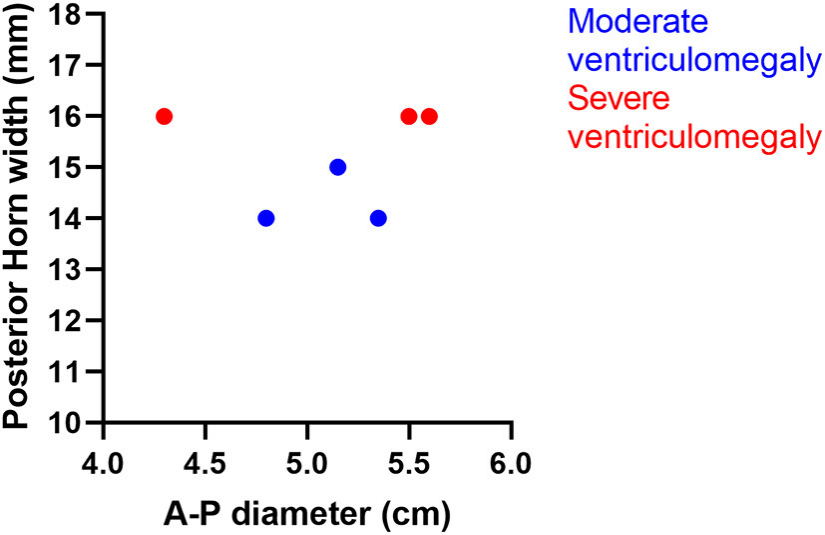
Distribution of the cases by the anterior–posterior diameter and the width of the posterior horn.

As described in the section regarding Methods, the dissected specimens were classified in two groups (Figure 2, Table 3): the moderate VM group including 3 of the patients (Patient 2, with 18 GW, and Patients 3 and 4 with 19 GW), with the atrial diameter between 13 and 15 mm and the severe VM group including the other 3 patients (Patient 1 with 17 GW, Patient 5 with 21 GW, and Patient 6 with 22 GW), with the atrial diameter over 15 mm. The individual findings from the dissection of each specimen were subsequently described in order, starting with the moderate VM group.

**Table 3:** Distribution of the patients into their specific groups.

**Moderate ventriculomegaly**			**Severe ventriculomegaly**		
**Patient's ID**	**Gestational age**	**Atrial diameter**	**Patient's ID**	**Gestational age**	**Atrial diameter**
Patient 2	18 GW	15 mm	Patient 1	17 GW	16 mm
Patient 3	19 GW	14 mm	Patient 5	21 GW	16 mm
Patient 4	19 GW	14 mm	Patient 6	22 GW	16 mm

### Moderate ventriculomegaly

**Figure 3:**
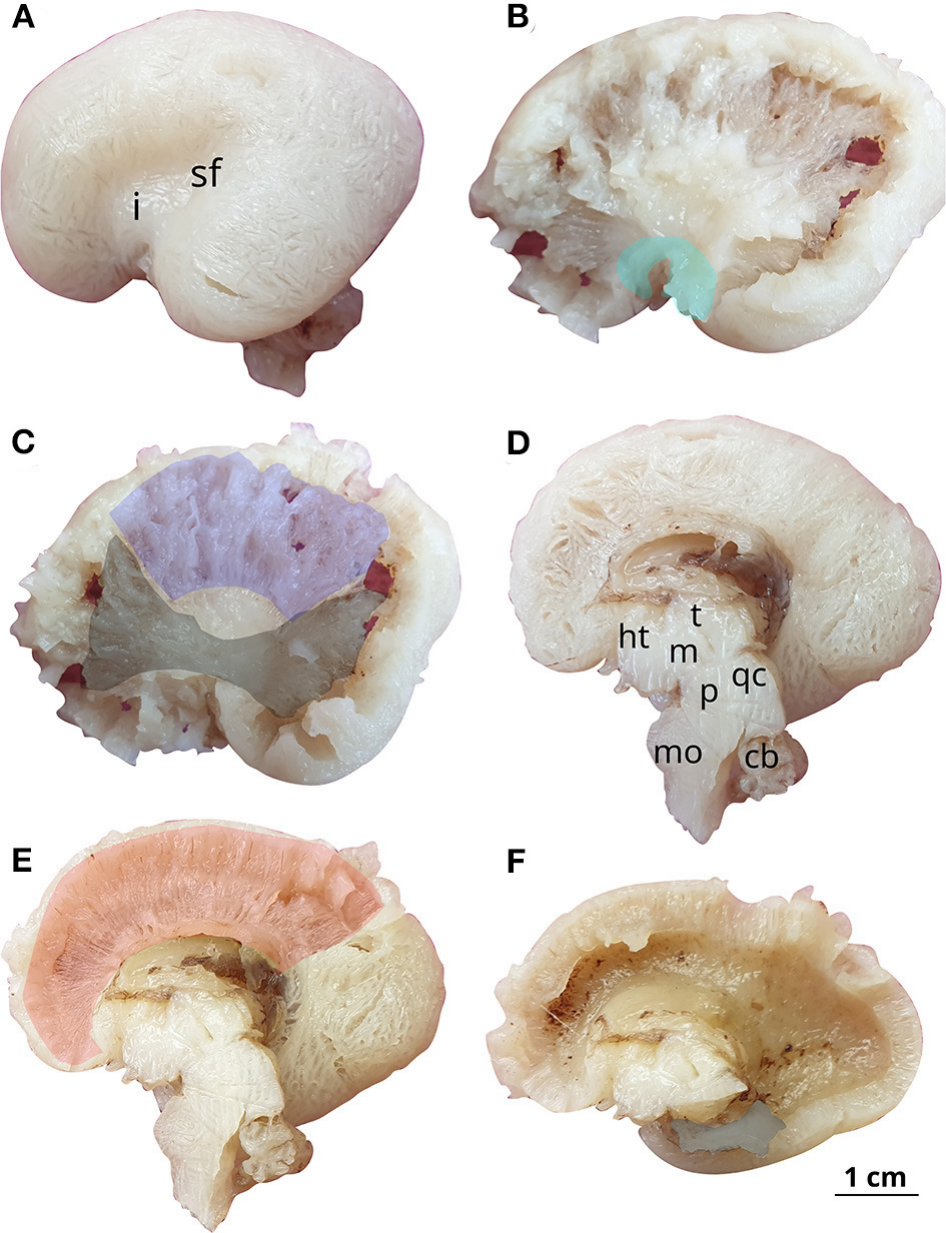
White matter tracts in the 18 GW brain. Successive latero-medial **(A–C)** and medio-lateral **(D–F)** dissection steps. Outlined white matter tracts, uncinate fasciculus (cyan), internal capsule (blue), inferior fronto-occipital fasciculus (black), corpus callosum (red), fornix (gray). i, insula; sf, sylvian fissure; ht, hypothalamus; t, thalamus; m, mesencephalon; p, pons; qc, quadrigeminal colliculi; mo, medulla oblongata; cb, cerebellum.

The youngest brain included in this category was the 18 GW brain (Patient 2), which had an A-P diameter of 5.15 cm. The diameter of the lateral ventricle measured at the level of atria was 15 mm, falling into the upper limit of the mild ventriculomegaly class. The external surface was globular, smooth, firm, and not easily deformable. The lateral fissure and insular cortex were to some extent identifiable, but other sulci were not visible at this stage. Following the dissection of the fibers, three commissural tracts were identified—the corpus callosum, fornix, and anterior commissure. The corpus callosum was well-defined, the fornix was fragmented at the moment of the dissection, and the anterior commissure was thin and difficult to highlight in our pictures. On the matter of association fibers, we were able to identify the uncinate and the inferior fronto-occipital fasciculi (Figure 3). In addition to that, this was the youngest brain on which the internal capsule could be clearly identified. The fibers discovered, although few in number, were individualized from one another.

**Figure 4:**
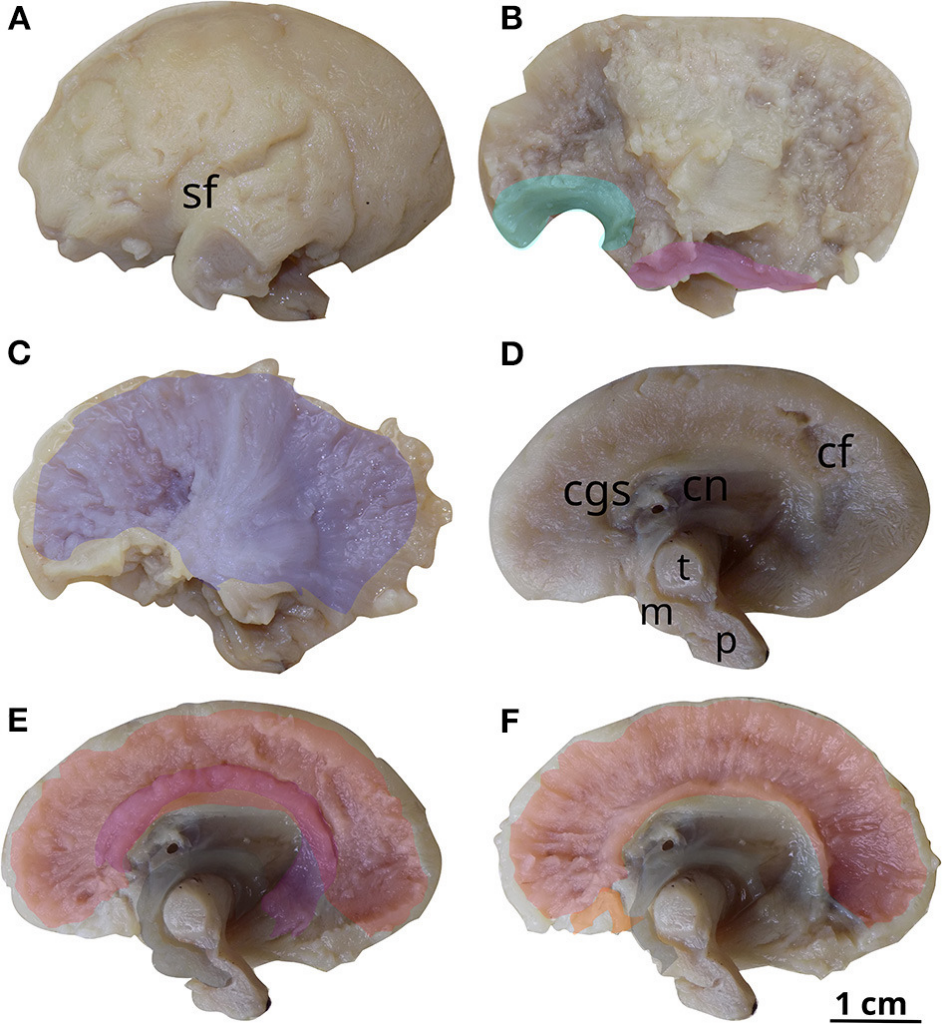
White matter tracts in the first 19 GW brain (Case 3). Successive latero-medial **(A–C)** and medio-lateral **(D–F)** dissection steps. Outlined white matter tracts, uncinate fasciculus (cyan), inferior longitudinal fasciculus (magenta), internal capsule (blue), cingulum (purple), corpus callosum (red), fornix (gray), anterior commissure (orange). sf, sylvian fissure; cgs, cingulate sulcus; cn, caudate nucleus; cf, calcarine fissure; t, thalamus; m, mesencephalon; p, pons.

Although there were two 19 GW specimens included in the study, the results found on the two dissections had some dissimilarities:

The first 19 GW brain was 4.8 cm in A-P diameter. The grade of VM was moderate, which was determined by the diameter of the atrium of 14 mm. The brain had an oval shape, resembling that of the adult brain. It was very fragile, very easily deformable, having a water balloon-like sensation to the touch. The Sylvian fissure could be observed, but the other sulci were just starting to develop. Regarding fiber dissection, the results were similar to the previous brain on the matter of commissural and projection tracts. Slight differences were identified concerning the association fasciculi—the uncinate, inferior longitudinal fasciculus, and cingulum being discovered on this brain (Figure 4).

**Figure 5:**
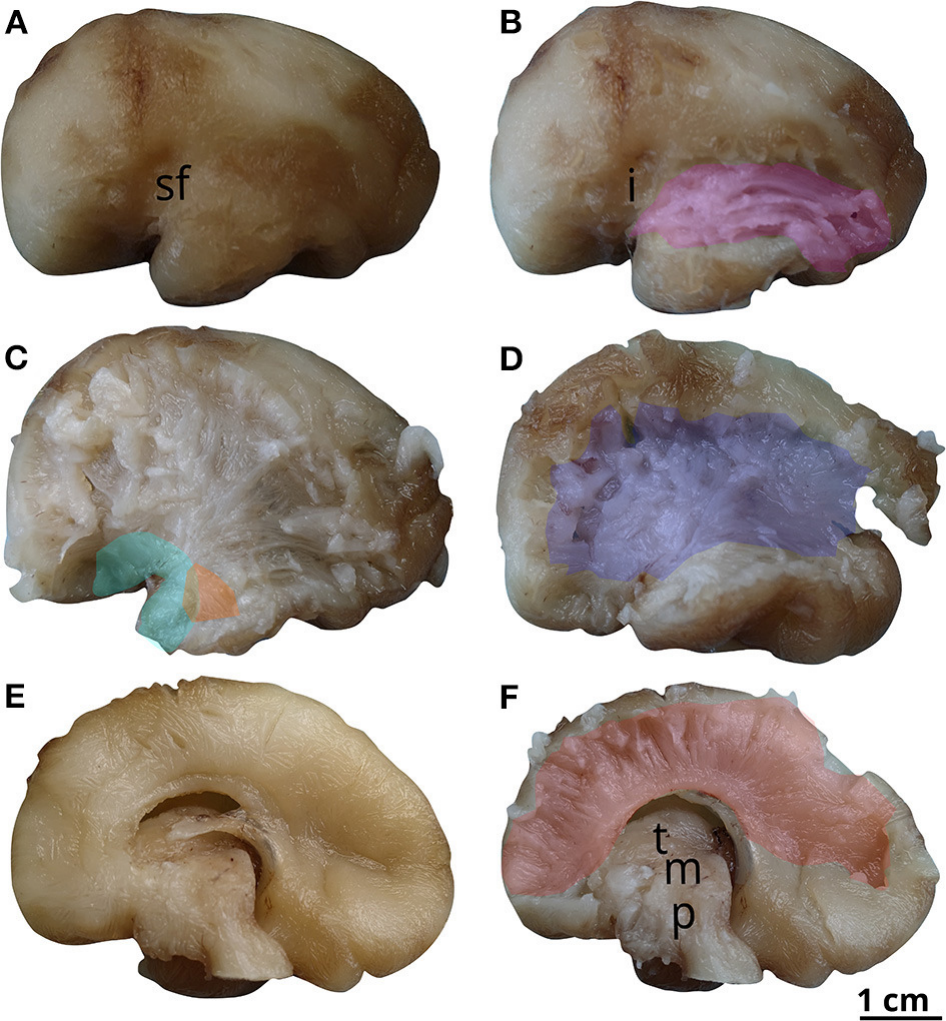
White matter tracts in the second 19 GW brain (Case 4). Successive latero-medial **(A–D)** and medio-lateral **(E, F)** dissection steps. Outlined white matter tracts, inferior longitudinal fasciculus (magenta), uncinate fasciculus (cyan), anterior commissure (orange), internal capsule (blue), corpus callosum (red), fornix (gray). Sf, sylvian fissure; t, thalamus ; m, mesencephalon; p, pons.

The second 19 GW brain was 5.35 cm in A-P diameter and had a moderate degree of VM, quantified by the diameter of the lateral ventricles measured on the ventricular atrium at 14 mm. The brain had an oval shape, a smooth surface, and a fair grade of deformability. The lateral fissure, central, and parieto-occipital sulci were able to be recognized, but the other sulci were not visible. The same tracts as in the other 19 GW brain could be identified (Figure 5).

A common finding in these two brains was the aspect of the uncinate and the inferior longitudinal fasciculus, which were thicker and better individualized than at 18 weeks.

### Severe ventriculomegaly

**Figure 6:**
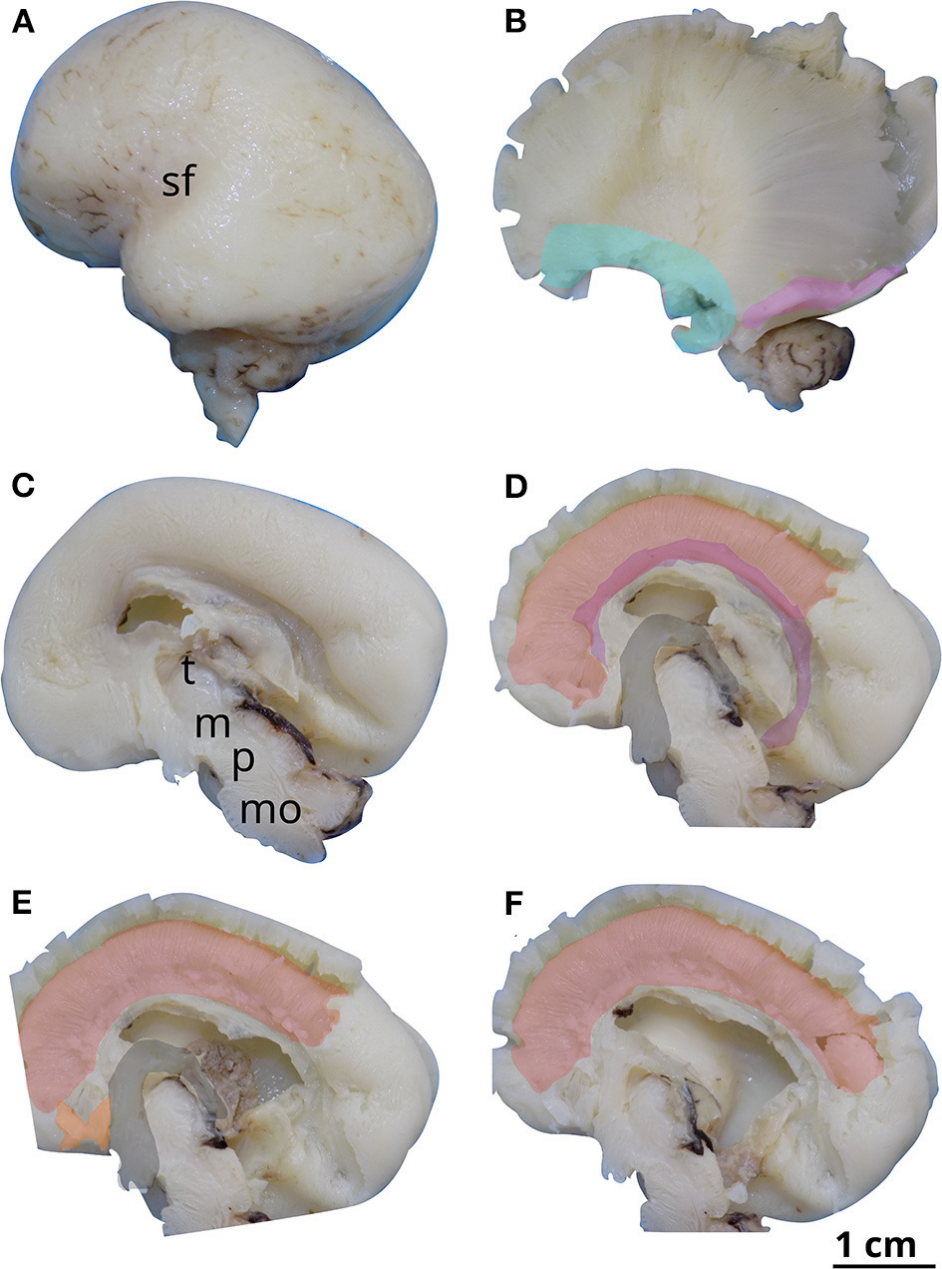
White matter tracts in the 17 GW brain. Successive latero-medial **(A, B)** and medio-lateral **(C–F)** dissection steps. Outlined white matter tracts, uncinate fasciculus (cyan), inferior longitudinal fasciculus (magenta), cingulum (purple), corpus callosum (red), fornix (gray), anterior commissure (orange). sf, sylvian fissure; t, thalamus; m, mesencephalon; p, pons; mo, medulla oblongata.

The youngest/smallest brain included in our study was only 17 GW and had a 4.3 cm in anterior–posterior diameter. The diameter of the lateral ventricle assessed at the atrium level was 16 mm, thus belonging to the severe ventriculomegaly group. The external appearance was smooth, globular, and firm. The sulci were not visible, except for the lateral fissure, which was starting to show. We were able to identify three association fasciculi—the uncinate fasciculus, the inferior longitudinal fasciculus, and the cingulum, as shown in Figure 6. The fibers were still thin and difficult to differentiate from one another. We were also able to observe the three commissural tracts—the anterior commissure, corpus callosum, and fornix. As for the projection fibers, the internal capsule could not be visualized at that time.

**Figure 7:**
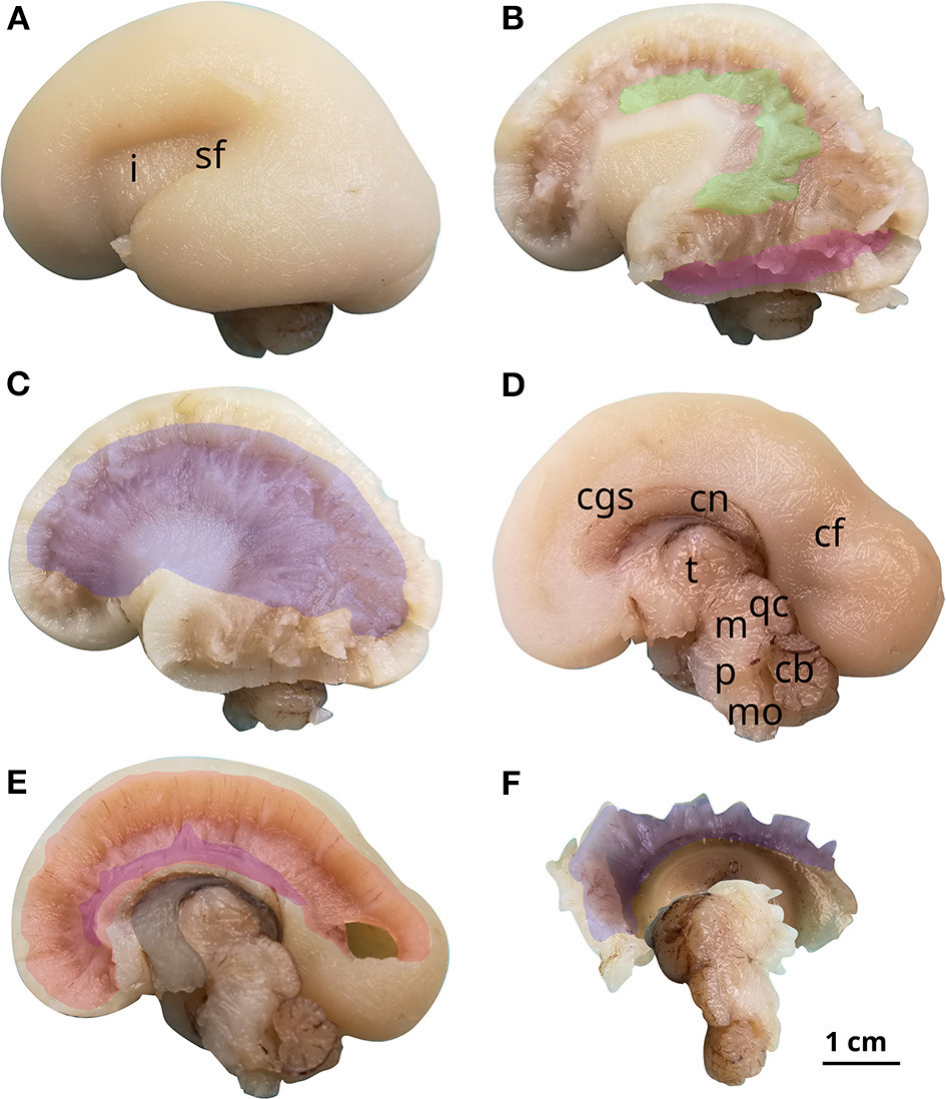
White matter tracts in the 21 GW brain. Successive latero-medial **(A–C)** and medio-lateral **(D–F)** dissection steps. Outlined white matter tracts, superior longitudinal fasciculus (green), inferior longitudinal fasciculus (magenta), internal capsule (blue), cingulum (purple), corpus callosum (red), fornix (gray). i, insula; sf, sylvian fissure; cgs, cingulate sulcus; cn, caudate nucleus; cf, calcarine fissure; t, thalamus; m, mesencephalon; p, pons; mo, medulla oblongata; qc, quadrigeminal colliculi; cb, cerebellum.

The 21 GW brain had an A-P diameter of 5.6 cm, and a severe degree of VM, quantified by measurements at the atria (16 mm) of the lateral ventricles. Its external appearance was paradoxically similar to the one found on the 18 GW brain, with a smooth, slightly oval surface, and very few sulci. The Sylvian fissure was very easily recognized, and the parieto-occipital sulcus was shallow and the other sulci could not be seen. With regard to the association fibers, the cingulum and the superior and inferior longitudinal fasciculus were able to be distinguished (Figure 7). This is the youngest brain on which we were able to identify the superior longitudinal fasciculus although not fully organized at this age. The three commissural tracts and the internal capsule could be exposed as well.

**Figure 8:**
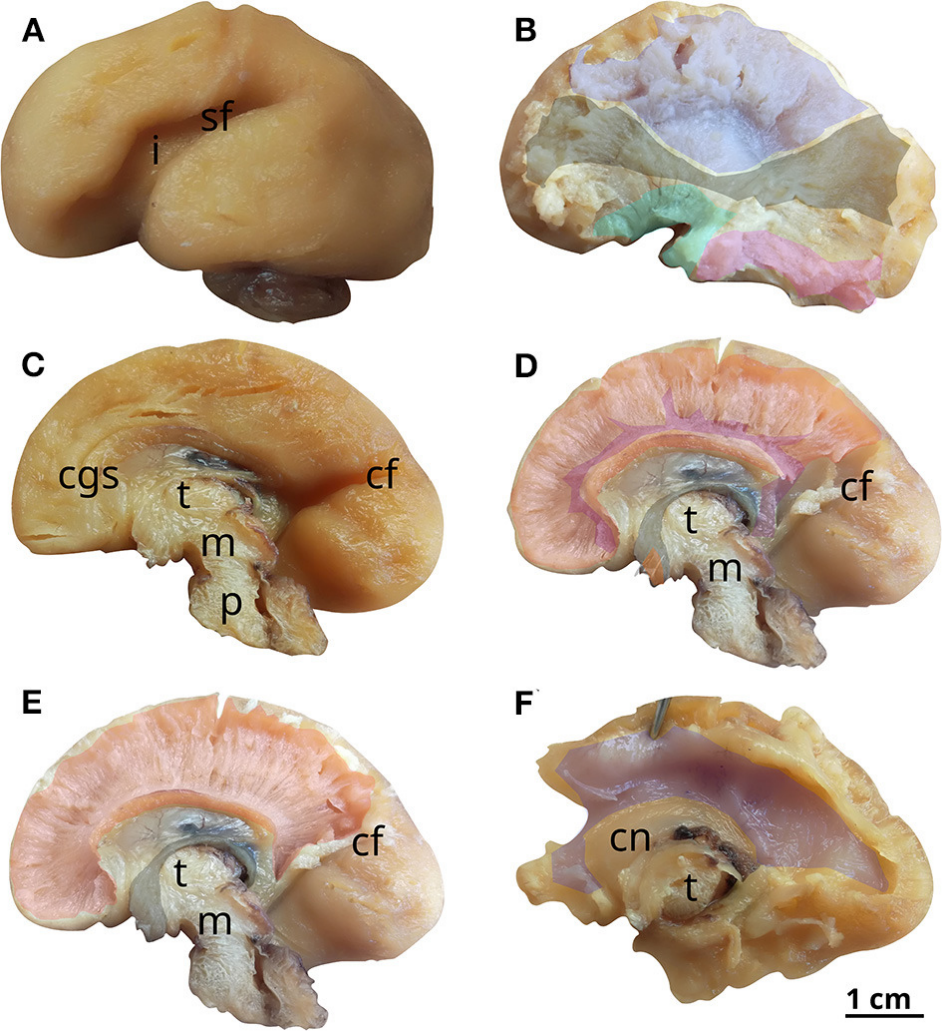
White matter tracts in the 22 GW brain. Successive latero-medial **(A, B)** and medio-lateral **(C–F)** dissection steps. Outlined white matter tracts, internal capsule (blue), uncinate fasciculus (cyan), inferior longitudinal fasciculus (magenta), inferior fronto-occipital fasciculus (black), cingulum (purple), corpus callosum (red), fornix (gray), anterior commissure (orange). i, insula; sf, sylvian fissure; cgs, cingulate sulcus; cf, calcarine fissure; t, thalamus; m, mesencephalon; p, pons; cn, caudate nucleus.

The oldest brain (22 GW) had a slightly smaller A-P diameter compared (5.5 cm) to the younger previous brain and a severe grade of ventriculomegaly, objectified by the 16 mm measured at the level of the atrium. The exterior aspect was very similar to that of the 19 GW brain, with an ovular, easily deformable surface. The severe VM gave the brain a water balloon-like sensation to the touch. The lateral fissure and the occipito-parietal sulcus could be well-observed and the central sulcus could also be identified, but the others were still not visible. The insular cortex was not yet covered. On dissection of the fibers, among the association fasciculi, the cingulum, the inferior longitudinal, inferior fronto-occipital, and uncinate fasciculi could be shown. The three commissural tracts and the internal capsule could also be observed (Figure 8).

## Discussion

### White matter development in VM compared to normal brains

For the purpose of comparing our results with normal brain specimens, we analysed the existing literature treating fetal brain architecture, by means of MRI, DTI, and histology.

First, looking at the exterior surface of the normal brains pictured by Huang et al. (2009), we could observe a smooth 17 GW brain, with only the slightest indication of a Sylvian fissure (similar appearance to our specimen with VM). Moving next to the 19 GW brain, we could observe the lateral, central and calcarine sulci on a brain with a more mature shape than that of the previous one. These findings were similar to that of our specimens, with the exception of a softer consistency found in our fetal brains due to the excessive accumulation of CSF in the ventricles. Finally, the 21 GW brain described in this piece of literature had a mature oval shape with a well-defined Sylvian fissure and a slight indication of the central and calcarine sulci. Again, we have a strong correlation with the findings from our dissection. Therefore, we can assume that the exterior aspect and the process of gyration are not severely affected by VM.

Further focusing on the white matter architecture, we analysed the results provided by Ouyang et al. (2015) and Huang and Vasung (2014), who had studied the aspects of white matter tracts in non-pathological brains from 14 and 13 GW to adulthood. Both studies systematized their findings by describing each fascicle at every gestational age studied. Starting with the limbic pathways, they found that both the fornix and cingulum are present at 17 GW and their development does not change significantly at 19 GW. As these tracts can be observed on our pathological specimens during our dissections, we can hypothesize that their development is not heavily affected by ventriculomegaly. With regard to the commissural tracts, researchers described the corpus callosum and anterior commissure identifiable as early as 14 GW (Ouyang et al., 2015), with the anterior part of the corpus callosum visible at 17 GW. They thus noted an anterior to posterior maturation of the tract. In comparison, in our study, we could clearly observe both the anterior and posterior aspects of the corpus callosum at all gestational ages studied, with a predominance of the anterior part at 17 GW. Regarding the association fasciculi, similar to our findings, the studies describe the aspects of the inferior longitudinal and uncinate fasciculi even before 17 GW and the ganglionic eminence present at 17, 19, and 21 GW and absent before birth. In addition, they mentioned that the superior longitudinal fasciculus could not be individualized until the last weeks of gestation. We were able to highlight the SLF at 21 GW but did not take into consideration the ganglionic eminence. Thereby, it is possible that the tract that we identified may have been the ganglionic eminence. Finally, the conformation and development of the internal capsule were thought to be similar to that of the corpus callosum, with its first appearance at 13 GW (Huang and Vasung, 2014) and an anterior to posterior maturation. In our dissections, we were only able to identify this tract from 18 GW on with the fibers still fragile at 19.

Given this comparison between normal and pathological brains, it can be concluded that at the same gestational age, there are no major differences in white matter architecture between the two groups. The most important observation on the VM brains is the inconsistency of fiber tract identification with increasing gestational age. The SLF was highlighted on the 21 GW specimen, but not on the 22 GW; the IFOF was distinguished on the 18 GW brain, but not identified neither on the two 19 GW, nor on the 21; the ILF was not visible on the 18 GW even though it was present on the 17 GW brain and the UF was unexpectedly unidentifiable on the 22 GW.

### Underlying mechanisms for white matter changes in VM

Ventriculomegaly can be caused by impaired cerebral spinal fluid circulation, or it may be the result of other primary brain malformations, destructive processes, or genetic syndromes (Etchegaray et al., 2020). White matter defects associated with VM can either result from the pressure exerted by the enlarged ventricles, or be a manifestation of the primary pathology. The overall aspect of the six pathological brains included in the study suggests that structural changes were mainly caused by the pressure exerted by the cerebrospinal fluid of the enlarged ventricles. The ventricular pressure acted directly by displacing the fascicles in direct contact (the fornix, uncinate fasciculus, and inferior longitudinal fasciculus) and converting the dorsal surface of the corpus callosum from a concave shape to a convex one. In addition to that, we observed the compression of the internal capsule and the association fibers situated laterally from the ventricle. These tracts seem to be indirectly affected. Experimental hydrocephalus studies in animal models showed that the subventricular zone is disrupted by the elevated pressure causing neurogenesis and glial cell developmental impairments which in turn may contribute to anatomical defects (di Curzio et al., 2013; Li et al., 2014). On the other hand, subventricular zone dysfunction can itself be responsible for VM as well as for neurodevelopmental changes (Rodríguez and Guerra, 2017; Ito et al., 2021).

In addition to mechanical displacement and neural proliferation defects, secondary modifications of white matter architecture could play a role. The brain has the ability, through neuroplasticity, to adapt to pathology by reorganizing its structure, functions, and connections. Proof of this can be found through the parallel between the normal and ventriculomegalic brains with similar A–P diameters (there was no great delay in the shaping and growth of the pathological brains) and comparable tract findings (at the same gestational age, the majority of the tracts—although with an altered aspect—could be discovered).

### Technical considerations

The use of the Klingler technique allowed us to directly visualize the tracts in relation to the dilated ventricles. Compared to tractography which is significantly affected by the increased volume of intracranial liquid, this aspect had no influence on the preparation of the specimens for fiber dissection.

Possible limitations in the ability of the fiber dissection technique to reveal all the tracts that are present in each brain specimen should be taken into consideration. As a general trend, the fibers distinguished on the hydrocephalic specimens were thinner, more fragile, and more difficult to individualize. The normal arborization of the corpus callosum and the internal capsule faded due to the infiltration of the fluid present in greater quantity, providing an edematous appearance. The same phenomenon occurs concerning the bundles of fibers that form the association tracts, making their trajectory more difficult to follow. The severity of the VM also influenced the accessibility of tracts by dissection. The compression was caused by the enlarged compacted various layers of fiber tracts, rendering them difficult to separate from one another. On the other hand, some tracts were anatomically more accessible to dissection (uncinate fasciculus), making them more likely to be detected in every specimen.

### Clinical relevance of fetal VM

The architectural changes identified in this study should encourage more extensive use of tractographic reconstructions in the *in utero* MRI assessment of fetal VM, which the current literature is lacking at this time. Conventional MRI may not consistently register this particular kind of change.

Many studies have focused on the structural modifications and clinical anomalies found in children antenatally diagnosed with VM.

Regarding the structural modifications, the evolution of the lateral ventricles, gray matter, and white matter was studied postnatally and in the first years of life. Lyall et al. (2012) noted the persistence of the enlargement of the lateral ventricles at 1 and 2 years. Gilmore et al. (2008) studied the gray and white matter by means of neonatal MRI and discovered that the intracranial volume and the volume of the gray matter were increased. Similar results were obtained by Kyriakopoulou et al. (2014) in their study on fetuses between 22 and 37.3 GW. An explanation for this phenomenon could be found in the formation of neural progenitors on the periventricular zone.

The white matter was not significantly modified with regard to absolute volume but was considered reduced in relation to the enlarged intracranial volume.

There are a considerable number of studies in the literature that attempt to debate the impact of VM on postnatal, infancy, and childhood neurodevelopment. From small deficits (Leitner et al., 2009; Lyall et al., 2012), some studies discovered even predisposition to neuropsychiatric diseases (Bromley et al., 1991; Hertzberg et al., 1994; Patel et al., 1994; Bloom et al., 1997; Sadock et al., 2007; Leitner et al., 2009).

Regarding the prevalence of developmental delay, we must take into consideration the degree of VM and the possible associated malformations of the analysed specimens. Gaglioti et al. (2005) categorized their cohort into three groups and found that the neurodevelopmental outcome was normal in 93% of the mild, 75% of the moderate, and 62.5% of the severe VM group. They also observed a significantly higher rate of associated malformations at a higher grade of VM (60% in the severe VM group); results previously observed by Vergani et al. (1998) noted an important difference in the prevalence of associated malformations between the mild and moderate VM groups. Some authors even consider the mild isolated VM a physiological variation (Signorelli et al., 2004; Giorgione et al., 2022), considering the small rate of neurodevelopment delay [between 3 and 9% (Vergani et al., 1998; Pilu et al., 1999; Falip et al., 2007; Pagani et al., 2014)], the good recovery prospects with specific therapy at school age (Sadan et al., 2007; Colitto et al., 2009; Atad-Rapoport et al., 2015), and the natural decrease in size of the ventricles after birth (Nelson et al., 2003). The prognosis was not influenced by the moment of diagnosis during pregnancy or by the evolution of VM. Falip et al. (2007) noted similar outcomes between groups with progressive, stable, or regressive VM.

Unfortunately, all cases included in our study had moderate to severe VM and a various number of associated malformations. Under these conditions, a poor prognosis is very likely. It is important to mention that for the statistical outcome numbers quoted above to be relevant, associated pathologies must be very carefully excluded through cerebral MRI, echocardiography, infectious markers and antibodies, karyotype, and even prenatal CGH-array in some cases (in search for microdeletions). Moreover, it is essential to correctly assess the grade of VM. For this purpose, bilateral measurement is indicated (Garel and Alberti, 2006; Falip et al., 2007). As demonstrated by Yaniv et al. (2016), changes in brain diffusivity in specific regions have been found depending on the form of VM.

## Conclusion

Displaced or absent white matter tracts can be identified in brains with VM by the use of stepwise tridimensional fiber dissection protocols. The findings tended to correlate with the severity of the ventricle enlargement but varied from one specimen to another and from one tract to another. In comparison to normal brain specimens, the tracts displayed only slightly different aspects or moments of appearance. These postmortem results should encourage more extensive use of *in utero* tractography to better characterize the neurodevelopmental delays in order to increase the accuracy of prognosis in cases of fetal ventriculomegaly.

## Data availability statement

The original contributions presented in the study are included in the article/supplementary material, further inquiries can be directed to the corresponding author.

## Ethics statement

The studies involving human participants were reviewed and approved by Ethics Committee, “Iuliu Haţieganu” University of Medicine and Pharmacy, Cluj-Napoca, Romania. The patients/participants provided their written informed consent to participate in this study. Written informed consent was obtained from the individual(s) for the publication of any potentially identifiable images or data included in this article.

## Author contributions

RP, SS, BS, RC, FS, ISF, AF, CA, and C-MM: study design. SS, RC, CA, and FS: brain collection. BH, MM, ABo, and RP: dissections. RP and BH: image processing. BH, RP, MM, ABo, ABu, and SS: manuscript preparation. SS, RP, BH, MM, ABo, ABu, BS, RC, ISF, AF, FS, and C-MM: manuscript review. All authors contributed to the article and approved the submitted version.
